# Supra­molecular architecture in a co-crystal of the N(7)—H tautomeric form of *N* ^6^-benzoyl­adenine with adipic acid (1/0.5)

**DOI:** 10.1107/S2056989016007581

**Published:** 2016-05-13

**Authors:** Robert Swinton Darious, Packianathan Thomas Muthiah, Franc Perdih

**Affiliations:** a School of Chemistry, Bharathidasan University, Tiruchirappalli 620 024, Tamilnadu, India; b Faculty of Chemistry and Chemical Technology, University of Ljubljana, Večna pot 113, PO Box 537, SI-1000 Ljubljana, Slovenia

**Keywords:** crystal structure, *N*^6^-benzoyl­adenine, adipic acid, hydrogen bond, supra­molecular sheet, π–π stacking, co-crystal

## Abstract

The asymmetric unit of the title co-crystal, C_12_H_9_N_5_O·0.5C_6_H_10_O_4_, consists of one mol­ecule of *N* ^6^-benzoyl­adenine (BA) and one half-mol­ecule of adipic acid (AA), the other half being generated by inversion symmetry. The dihedral angle between the adenine and phenyl ring planes is 26.71 (7)°. The *N* ^6^-benzoyl­adenine mol­ecule crystallizes in the N(7)—H tautomeric form with three non-protonated N atoms. This tautomeric form is stabilized by intra­molecular N—H⋯O hydrogen bonding between the carbonyl (C=O) group and the N(7)—H hydrogen atom on the Hoogsteen face of the purine ring, forming an *S*(7) ring motif. The two carboxyl groups of adipic acid inter­act with the Watson–Crick face of the BA mol­ecules through O—H⋯N and N—H⋯O hydrogen bonds, generating an *R*_2_^2^(8) ring motif. The latter units are linked by N—H⋯N hydrogen bonds, forming layers parallel to (10-5). A weak C—H⋯O hydrogen bond is also present, linking adipic acid mol­ecules in neighbouring layers, enclosing *R* ^2^_2_(10) ring motifs and forming a three-dimensional structure. C=O⋯π and C—H⋯π inter­actions are also present in the structure.

## Abstract 2

The supra­molecular architecture in a co-crystal of N(7)—H tautomeric form of *N* ^6^-benzoyl­adenine-adipic acid (1/0.5) is reported. The typical C=O⋯π and C—H⋯π inter­actions are also present in this structure.

## Chemical context   

Adipic acid has been widely used in controlled-release formulations of many drugs and food additives (Roew *et al.*, 2009 ▸). *N* ^6^-benzoyl­adenine is a synthetic analogue of a group of naturally occurring *N* ^6^-substituted adenines having plant-growth-stimulating activity (cytokinins) (McHugh & Erxleben, 2011 ▸). A number of co-crystals involving adipic acid have been reported in the literature (Lemmerer *et al.*, 2012 ▸; Lin *et al.*, 2012 ▸; Matulková *et al.*, 2014 ▸; Thanigaimani *et al.*, 2012 ▸). This paper deals with a co-crystal formed between *N* ^6^-benzoyl­adenine and adipic acid (I).
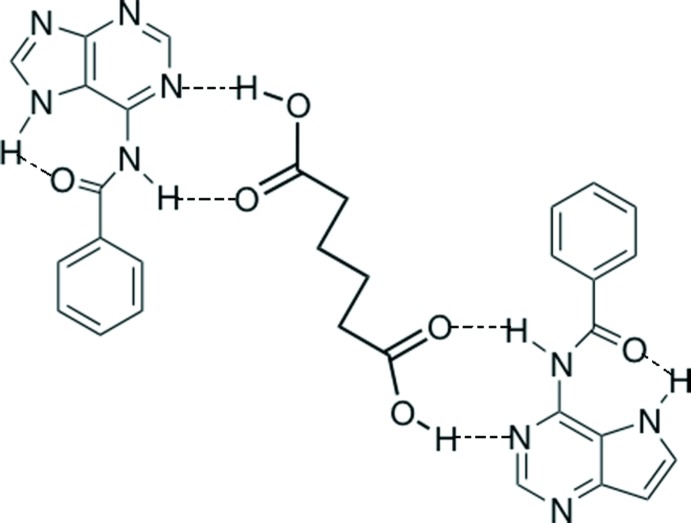


## Structural commentary   

The asymmetric unit of (I) contains one *N* ^6^-benzoyl­­adenine (BA) mol­ecule and a half-mol­ecule of adipic acid (AA). As evident from the angles at N7 [C8—N7—C5 = 106.82 (11)°] and N9 [C8—N9—C4 = 103.90 (11)°], the *N* ^6^-benzoyl­adenine moiety exists in the N(7)—H tautomeric form with non-protonated N1, N3 and N9 atoms. In addition, the C8—N7 bond [1.3415 (17) Å)] is longer than C8—N9 [1.3175 (19) Å]. These values are similar to those in neutral *N* ^6^-benzoyl­adenine (Raghunathan & Pattabhi, 1981 ▸). An intra­molecular hydrogen bond in the Hoogsteen face between N7—H7 and the benzoyl oxygen atom O1 forms a *S*(7) ring motif. The dihedral angle between the adenine and phenyl ring plane is 26.71 (7)° and the C6—N6—C10—C11 torsion angle is 173.08 (14)°. The bond lengths and bond angles of AA are in the range of values reported (Srinivasa Gopalan *et al.*, 1999 ▸; 2000 ▸). The values for the torsion angles C18—C19—C19a—C18a [180.00 (13)°] and C17—C18—C19—C19a [–176.09 (14)°] indicate that the carbon chain of AA is fully extended.

In the crystal structures of *N* ^6^-benzyl­adenine (Raghunathan & Pattabhi, 1981 ▸), *N* ^6^-furfuryladenine (Soriano-Garcia & Parthasarathy, 1977 ▸), *N* ^6^-benzyl­adenine hydro­bromide (Umadevi *et al.*, 2001 ▸), *N* ^6^-furfuryladenine hydro­chloride (Stanley *et al.*, 2003 ▸), *N* ^6^-benzyl­adeninium *p*-toluene­sulfonate (Tamilselvi & Mu­thiah, 2011 ▸), *N* ^6^-benzyl­adeninium nitrate, *N* ^6^-benzyl­adeninium 3-hy­droxy picolinate (Nirmalram *et al.*, 2011 ▸) and the hydrate adduct of *N* ^6^-benzyl­adenine-5-sulfo­sali­cylic acid (Xia *et al.*, 2010 ▸), the *N* ^6^-substituent is distal to the N7 position, whereas in the crystal structures of *N* ^6^-benzoyl­adenine (Raghunathan *et al.*, 1983 ▸), *N* ^6^-benzoyl­adenine-3-hy­droxy­pyridinium-2-carboxyl­ate (1:1), *N* ^6^-benz­oyl­adenine-dl-tartaric acid (1:1) (Karthikeyan *et al.*, 2015 ▸), *N* ^6^-benzoyl­adeninium nitrate (Karthikeyan *et al.*, 2015 ▸) and the title compound, the *N* ^6^-substituent is distal to N1 and *syn* to adenine nitrogen atom N7. In the present structure, this may be attributed to the presence of the N7—H7⋯O1*A* intra­molecular hydrogen bond (Table 1 ▸).

## Supra­molecular features   

**Figure 1:**
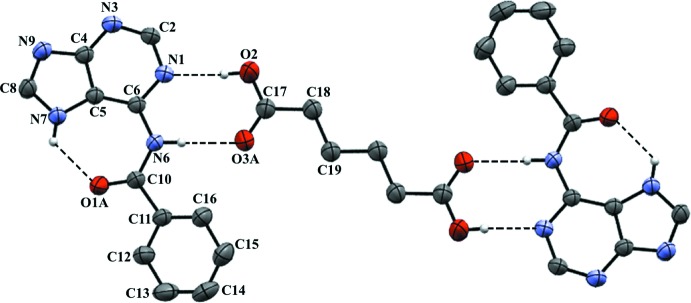
A *Mercury* (Macrae *et al.*, 2008 ▸) view of the title compound (I), showing the atom-numbering scheme. Disordered oxygen atoms are omitted for clarity. H atoms not involved in hydrogen bonding have been omitted for clarity. Unlabelled atoms are related by the symmetry operation 1 − *x*, 1 − *y*, −*z*.

**Figure 2:**
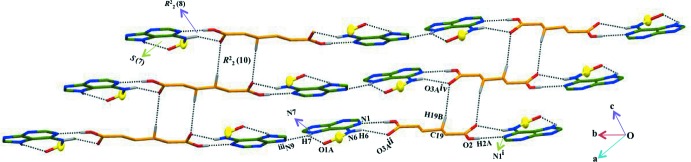
A view of the sheet-like supra­molecular architecture generated *via* C19—H19*B*⋯O3*A* hydrogen bonds (black dotted lines). Phenyl rings are indicated as yellow balls. H atoms not involved in hydrogen bonding have been omitted for clarity. Symmetry codes are as given in Table 1 ▸.

Each of the two carboxyl groups of adipic acid inter­acts with the Watson–Crick face (atoms N1 and N6) of the corres­ponding BA through O—H⋯N and N—H⋯O hydrogen bonds, generating an 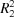
(8) ring motif (Fig. 1 ▸). Thus each adipic acid mol­ecule bridges two BA mol­ecules. The latter units are linked by N7—H7⋯N9^iii^ hydrogen bonds (Table 1 ▸) forming layers parallel to plane (10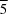). A weak C—H⋯O hydrogen bond (C19—H19*B*⋯O3*A* ^iv^) is also present (Table 1 ▸ and Fig. 2 ▸), linking adipic acid mol­ecules in neighbouring layers, enclosing 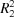
(10) ring motifs and forming a three-dimensional structure. Thus atom O3*A* functions as a bifurcated hydrogen-bond acceptor whereas N7—H is a bifurcated hydrogen-bond donor.

**Figure 3:**
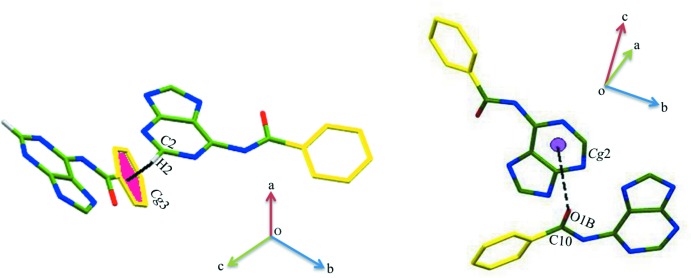
(*a*) A view of the C—H⋯π inter­action in compound (I). *Cg*3 is the centroid of the phenyl ring of the BA mol­ecule (symmetry code: *x*, −1 + *y*, *z*). (*b*) A view of the C=O⋯π inter­action. *Cg*2 is the centroid of the pyrimidine ring of the BA mol­ecule (symmetry code: 1 − *x*, 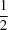 + *y*, 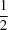 − *z*).

The crystal structure also features C2—H2⋯π inter­actions between purine and phenyl rings (Fig. 3 ▸ *a*) and C10=O1*B*⋯π inter­actions between the carbonyl oxygen O1*B* and the centroid of the (N1/C2/N3/C4/C5/C6) pyrimidine ring [O⋯centroid = 3.407 (10) Å; symmetry code: 1 − *x*, 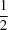 + *y*, 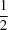 − *z*; Fig. 3 ▸ *b*] (Safaei-Ghomi *et al.*, 2009 ▸).

## Database survey   

The neutral mol­ecule *N* ^6^-benzoyl­adenine was reported by Raghunathan & Pattabhi (1981 ▸). Co-crystals have also been reported: *N* ^6^-benzoyl­adenine-3-hy­droxy­pyridinium-2-carb­oxyl­ate (1:1), *N* ^6^-benzoyl­adenine-dl-tartaric acid (1:1) (Karthikeyan *et al.*, 2015 ▸) and *N* ^6^-benzoyl­adeninium nitrate (Karthikeyan *et al.*, 2016 ▸). Similarly, co-crystals of adipic acid with pyrimidine derivatives [adenine (Byres *et al.*, 2009 ▸), caffeine (Bučar *et al.*, 2007 ▸), cytosine (Das & Baruah, 2011 ▸), bis-pyrimidine-amine-linked xylene spacer (Goswami *et al.*, 2010 ▸)] have also been reported.

## Synthesis and crystallization   

The title co-crystal was synthesized by mixing a DMF solution of *N* ^6^-benzoyl­adenine (30 mg) and adipic acid (19 mg) (total volume = 10 mL). The mixture was warmed in a water bath for 20 min. After cooling to room temperature, colourless plate-like crystals were collected from the mother liquor after a few days (m.p. 438 K).

## Refinement   

Crystal data, data collection and structure refinement details are summarized in Table 2 ▸. Atoms O1 and O3 are disordered over two positions with refined occupancy ratios of 0.57 (3):0.43 (3) and 0.63 (3):0.37 (3), respectively. Hydrogen atoms were readily located in difference Fourier maps and were subsequently treated as riding atoms in geometrically idealized positions, with C—H = 0.93 (aromatic) or 0.97 (methyl­ene), N—H = 0.86, and O—H = 0.82 Å, and with *U*_iso_(H) = *kU*_eq_(C,N,O), where *k* = 1.5 for hy­droxy and 1.2 for all other H atoms.

## Supplementary Material

Crystal structure: contains datablock(s) I. DOI: 10.1107/S2056989016007581/hg5474sup1.cif

Structure factors: contains datablock(s) I. DOI: 10.1107/S2056989016007581/hg5474Isup2.hkl

Supporting information file. DOI: 10.1107/S2056989016007581/hg5474Isup3.cml

CCDC reference: 1478504

Additional supporting information:  crystallographic information; 3D view; checkCIF report

## Figures and Tables

**Table 1:** Hydrogen-bond geometry (Å, °) *Cg* is the centroid of the C11–C16 phenyl ring.

*D*—H⋯*A*	*D*—H	H⋯*A*	*D*⋯*A*	*D*—H⋯*A*
O2—H2*A*⋯N1^i^	0.82	1.92	2.7327 (19)	175
N6—H6⋯O3*A* ^ii^	0.86	2.09	2.904 (11)	157
N7—H7⋯O1*A*	0.86	2.04	2.616 (16)	124
N7—H7⋯N9^iii^	0.86	2.17	2.9271 (17)	146
C19—H19*B*⋯O3*A* ^iv^	0.97	2.54	3.481 (11)	164
C2—H2⋯*Cg*3^v^	0.93	2.94	3.4611 (16)	117

Symmetry codes: (i) 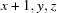; (ii) 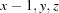; (iii) 
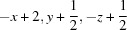
; (iv) 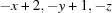; (v) 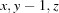.

**Table 2:** Experimental details

Crystal data	
Chemical formula	C_12_H_9_N_5_O·0.5C_6_H_10_O_4_
*M*_r_	312.31
Crystal system, space group	Monoclinic, *P*2_1_/*c*
Temperature (K)	293
*a*, *b*, *c* (Å)	6.1776 (4), 9.2296 (4), 25.7480 (15)
β (°)	97.117 (6)
*V* (Å^3^)	1456.76 (14)
*Z*	4
Radiation type	Mo *K*α
μ (mm^−1^)	0.10
Crystal size (mm)	0.60 × 0.60 × 0.40

Data collection	
Diffractometer	Agilent SuperNova Dual Source diffractometer with an Atlas detector
Absorption correction	Multi-scan (*CrysAlis PRO*; Agilent, 2013 ▸)
*T*_min_, *T*_max_	0.756, 1.000
No. of measured, independent and observed [*I* > 2σ(*I*)] reflections	9480, 3325, 2755
*R*_int_	0.020
(sin θ/λ)_max_ (Å^−1^)	0.649

Refinement	
*R*[*F* ^2^ > 2σ(*F* ^2^)], *wR*(*F* ^2^), *S*	0.045, 0.122, 1.05
No. of reflections	3325
No. of parameters	230
H-atom treatment	H-atom parameters constrained
Δρ_max_, Δρ_min_ (e Å^−3^)	0.25, −0.22

Computer programs: *CrysAlis PRO* (Agilent, 2013 ▸), *SUPERFLIP* (Palatinus & Chapuis, 2007 ▸), *SHELXL2014* (Sheldrick, 2015 ▸), *PLATON* (Spek, 2009 ▸) and *Mercury* (Macrae *et al.*, 2008 ▸).

## supplementary crystallographic information

### Crystal data

**Table:**

C_12_H_9_N_5_O·0.5C_6_H_10_O_4_	*F*(000) = 652
*M**_r_* = 312.31	*D*_x_ = 1.424 Mg m^−^^3^
Monoclinic, *P*2_1_/*c*	Mo *K*α radiation, λ = 0.71073 Å
*a* = 6.1776 (4) Å	Cell parameters from 4139 reflections
*b* = 9.2296 (4) Å	θ = 3.3–30.1°
*c* = 25.7480 (15) Å	µ = 0.10 mm^−^^1^
β = 97.117 (6)°	*T* = 293 K
*V* = 1456.76 (14) Å^3^	Prism, colorless
*Z* = 4	0.60 × 0.60 × 0.40 mm

### Data collection

**Table:**

Agilent SuperNova Dual Source diffractometer with an Atlas detector	3325 independent reflections
Radiation source: SuperNova (Mo) X-ray Source	2755 reflections with *I* > 2σ(*I*)
Mirror monochromator	*R*_int_ = 0.020
Detector resolution: 10.4933 pixels mm^-1^	θ_max_ = 27.5°, θ_min_ = 3.2°
ω scans	*h* = −8→7
Absorption correction: multi-scan (*CrysAlis PRO*; Agilent, 2013)	*k* = −11→11
*T*_min_ = 0.756, *T*_max_ = 1.000	*l* = −33→31
9480 measured reflections	

### Refinement

**Table:**

Refinement on *F*^2^	Hydrogen site location: mixed
Least-squares matrix: full	H-atom parameters constrained
*R*[*F*^2^ > 2σ(*F*^2^)] = 0.045	*w* = 1/[σ^2^(*F*_o_^2^) + (0.0541*P*)^2^ + 0.3295*P*] where *P* = (*F*_o_^2^ + 2*F*_c_^2^)/3
*wR*(*F*^2^) = 0.122	(Δ/σ)_max_ < 0.001
*S* = 1.05	Δρ_max_ = 0.25 e Å^−^^3^
3325 reflections	Δρ_min_ = −0.22 e Å^−^^3^
230 parameters	Extinction correction: *SHELXL2014* (Sheldrick, 2015), Fc^*^=kFc[1+0.001xFc^2^λ^3^/sin(2θ)]^-1/4^
0 restraints	Extinction coefficient: 0.0130 (18)

### Special details

**Table:**

Geometry. All esds (except the esd in the dihedral angle between two l.s. planes) are estimated using the full covariance matrix. The cell esds are taken into account individually in the estimation of esds in distances, angles and torsion angles; correlations between esds in cell parameters are only used when they are defined by crystal symmetry. An approximate (isotropic) treatment of cell esds is used for estimating esds involving l.s. planes.

### Fractional atomic coordinates and isotropic or equivalent isotropic displacement parameters (Å^2^)

**Table:**

	*x*	*y*	*z*	*U*_iso_*/*U*_eq_	Occ. (<1)
O1A	0.7104 (15)	0.6427 (14)	0.1669 (8)	0.094 (4)	0.57 (3)
O1B	0.6582 (19)	0.6621 (6)	0.1877 (4)	0.054 (2)	0.43 (3)
N1	0.3308 (2)	0.27081 (12)	0.16588 (5)	0.0415 (3)	
N3	0.5648 (2)	0.09828 (13)	0.21438 (5)	0.0477 (3)	
N6	0.39990 (19)	0.51100 (12)	0.15270 (5)	0.0391 (3)	
H6	0.2702	0.5109	0.1361	0.047*	
N7	0.85319 (19)	0.42515 (12)	0.22672 (5)	0.0402 (3)	
H7	0.8790	0.5157	0.2226	0.048*	
N9	0.9054 (2)	0.19667 (13)	0.25397 (5)	0.0470 (3)	
C2	0.3848 (3)	0.13827 (15)	0.18545 (6)	0.0468 (4)	
H2	0.2811	0.0660	0.1774	0.056*	
C4	0.7054 (2)	0.20808 (14)	0.22480 (5)	0.0389 (3)	
C5	0.6683 (2)	0.35149 (13)	0.20707 (5)	0.0352 (3)	
C6	0.4717 (2)	0.38085 (13)	0.17619 (5)	0.0347 (3)	
C8	0.9855 (3)	0.32896 (15)	0.25376 (6)	0.0451 (4)	
H8	1.1219	0.3535	0.2709	0.054*	
C10	0.5100 (3)	0.63803 (16)	0.15283 (7)	0.0493 (4)	
C11	0.4104 (2)	0.75951 (14)	0.11985 (6)	0.0412 (3)	
C12	0.5550 (3)	0.86137 (17)	0.10534 (7)	0.0544 (4)	
H12	0.7028	0.8527	0.1173	0.065*	
C13	0.4831 (3)	0.97575 (19)	0.07333 (8)	0.0627 (5)	
H13	0.5825	1.0423	0.0630	0.075*	
C14	0.2660 (3)	0.99109 (19)	0.05686 (7)	0.0628 (5)	
H14	0.2168	1.0681	0.0353	0.075*	
C15	0.1208 (3)	0.8931 (2)	0.07213 (8)	0.0655 (5)	
H15	−0.0274	0.9047	0.0611	0.079*	
C16	0.1909 (3)	0.77622 (18)	0.10389 (7)	0.0538 (4)	
H16	0.0908	0.7102	0.1142	0.065*	
O2	0.9399 (2)	0.25032 (13)	0.10377 (6)	0.0694 (4)	
H2A	1.0565	0.2620	0.1223	0.104*	
O3A	1.0228 (15)	0.4644 (11)	0.0753 (5)	0.072 (2)	0.63 (3)
O3B	0.951 (3)	0.4828 (6)	0.0985 (9)	0.070 (5)	0.37 (3)
C17	0.8870 (3)	0.36824 (17)	0.07825 (6)	0.0494 (4)	
C18	0.6762 (3)	0.36172 (16)	0.04285 (6)	0.0491 (4)	
H18A	0.5619	0.3312	0.0631	0.059*	
H18B	0.6882	0.2888	0.0162	0.059*	
C19	0.6100 (3)	0.50345 (16)	0.01626 (6)	0.0494 (4)	
H19A	0.6069	0.5781	0.0427	0.059*	
H19B	0.7188	0.5308	−0.0060	0.059*	

### Atomic displacement parameters (Å^2^)

**Table:**

	*U*^11^	*U*^22^	*U*^33^	*U*^12^	*U*^13^	*U*^23^
O1A	0.047 (3)	0.085 (4)	0.138 (8)	−0.023 (2)	−0.033 (4)	0.071 (4)
O1B	0.052 (3)	0.028 (2)	0.073 (4)	−0.0097 (15)	−0.024 (2)	0.011 (2)
N1	0.0423 (6)	0.0333 (6)	0.0467 (7)	−0.0045 (5)	−0.0037 (5)	0.0046 (5)
N3	0.0559 (8)	0.0299 (6)	0.0536 (7)	−0.0047 (5)	−0.0074 (6)	0.0067 (5)
N6	0.0365 (6)	0.0308 (6)	0.0470 (6)	0.0002 (4)	−0.0071 (5)	0.0062 (5)
N7	0.0399 (6)	0.0269 (5)	0.0503 (7)	0.0015 (5)	−0.0079 (5)	0.0000 (5)
N9	0.0495 (7)	0.0310 (6)	0.0561 (7)	0.0051 (5)	−0.0108 (6)	0.0036 (5)
C2	0.0519 (9)	0.0325 (7)	0.0528 (8)	−0.0091 (6)	−0.0060 (7)	0.0062 (6)
C4	0.0444 (8)	0.0292 (6)	0.0412 (7)	0.0023 (5)	−0.0023 (6)	0.0017 (5)
C5	0.0381 (7)	0.0280 (6)	0.0383 (7)	0.0015 (5)	−0.0002 (6)	−0.0008 (5)
C6	0.0380 (7)	0.0290 (6)	0.0362 (6)	0.0006 (5)	0.0007 (6)	0.0015 (5)
C8	0.0423 (8)	0.0341 (7)	0.0550 (8)	0.0045 (6)	−0.0101 (7)	−0.0007 (6)
C10	0.0461 (8)	0.0357 (7)	0.0611 (9)	−0.0047 (6)	−0.0131 (7)	0.0124 (7)
C11	0.0472 (8)	0.0303 (6)	0.0441 (7)	0.0017 (6)	−0.0019 (6)	0.0044 (5)
C12	0.0510 (9)	0.0398 (8)	0.0710 (11)	−0.0011 (7)	0.0017 (8)	0.0131 (7)
C13	0.0716 (12)	0.0437 (9)	0.0746 (12)	0.0012 (8)	0.0155 (10)	0.0202 (8)
C14	0.0809 (13)	0.0479 (9)	0.0590 (10)	0.0163 (9)	0.0061 (9)	0.0205 (8)
C15	0.0542 (10)	0.0617 (11)	0.0771 (12)	0.0142 (9)	−0.0059 (9)	0.0204 (9)
C16	0.0464 (9)	0.0455 (8)	0.0672 (10)	0.0021 (7)	−0.0019 (8)	0.0146 (7)
O2	0.0568 (7)	0.0461 (6)	0.0963 (10)	−0.0026 (5)	−0.0264 (7)	0.0128 (6)
O3A	0.061 (3)	0.072 (2)	0.076 (4)	−0.026 (2)	−0.024 (3)	0.027 (2)
O3B	0.069 (5)	0.037 (2)	0.092 (8)	−0.006 (2)	−0.035 (5)	0.002 (2)
C17	0.0477 (9)	0.0428 (8)	0.0544 (9)	−0.0017 (7)	−0.0073 (7)	0.0029 (7)
C18	0.0472 (8)	0.0408 (8)	0.0558 (9)	−0.0017 (6)	−0.0071 (7)	−0.0016 (7)
C19	0.0481 (9)	0.0411 (8)	0.0554 (9)	−0.0023 (6)	−0.0070 (7)	0.0023 (7)

### Geometric parameters (Å, º)

**Table:**

O1A—C10	1.247 (6)	C12—C13	1.378 (2)
O1B—C10	1.221 (6)	C12—H12	0.9300
N1—C6	1.3424 (17)	C13—C14	1.363 (3)
N1—C2	1.3489 (17)	C13—H13	0.9300
N3—C2	1.3125 (19)	C14—C15	1.365 (3)
N3—C4	1.3401 (18)	C14—H14	0.9300
N6—C10	1.3551 (18)	C15—C16	1.390 (2)
N6—C6	1.3926 (16)	C15—H15	0.9300
N6—H6	0.8600	C16—H16	0.9300
N7—C8	1.3415 (17)	O2—C17	1.2923 (18)
N7—C5	1.3712 (17)	O2—H2A	0.8200
N7—H7	0.8601	O3A—C17	1.230 (4)
N9—C8	1.3175 (19)	O3B—C17	1.223 (6)
N9—C4	1.3684 (18)	C17—C18	1.495 (2)
C2—H2	0.9300	C18—C19	1.509 (2)
C4—C5	1.4096 (17)	C18—H18A	0.9700
C5—C6	1.3931 (18)	C18—H18B	0.9700
C8—H8	0.9300	C19—C19^i^	1.506 (3)
C10—C11	1.4924 (18)	C19—H19A	0.9700
C11—C16	1.376 (2)	C19—H19B	0.9700
C11—C12	1.380 (2)		
			
C6—N1—C2	119.23 (11)	C13—C12—H12	119.6
C2—N3—C4	112.56 (12)	C11—C12—H12	119.6
C10—N6—C6	127.77 (11)	C14—C13—C12	119.81 (17)
C10—N6—H6	116.0	C14—C13—H13	120.1
C6—N6—H6	116.2	C12—C13—H13	120.1
C8—N7—C5	106.82 (11)	C13—C14—C15	119.86 (15)
C8—N7—H7	126.7	C13—C14—H14	120.1
C5—N7—H7	126.5	C15—C14—H14	120.1
C8—N9—C4	103.90 (11)	C14—C15—C16	121.01 (16)
N3—C2—N1	128.29 (13)	C14—C15—H15	119.5
N3—C2—H2	115.9	C16—C15—H15	119.5
N1—C2—H2	115.9	C11—C16—C15	119.12 (16)
N3—C4—N9	124.79 (12)	C11—C16—H16	120.4
N3—C4—C5	124.74 (12)	C15—C16—H16	120.4
N9—C4—C5	110.47 (12)	C17—O2—H2A	109.5
N7—C5—C6	137.86 (12)	O3B—C17—O2	117.6 (6)
N7—C5—C4	104.56 (11)	O3A—C17—O2	120.5 (3)
C6—C5—C4	117.57 (12)	O3B—C17—C18	120.5 (3)
N1—C6—N6	113.77 (11)	O3A—C17—C18	122.7 (2)
N1—C6—C5	117.61 (11)	O2—C17—C18	114.98 (13)
N6—C6—C5	128.60 (12)	C17—C18—C19	114.09 (13)
N9—C8—N7	114.25 (12)	C17—C18—H18A	108.7
N9—C8—H8	122.9	C19—C18—H18A	108.7
N7—C8—H8	122.9	C17—C18—H18B	108.7
O1B—C10—N6	119.4 (5)	C19—C18—H18B	108.7
O1A—C10—N6	120.7 (5)	H18A—C18—H18B	107.6
O1B—C10—C11	120.0 (3)	C19^i^—C19—C18	112.99 (16)
O1A—C10—C11	117.6 (3)	C19^i^—C19—H19A	109.0
N6—C10—C11	118.50 (12)	C18—C19—H19A	109.0
C16—C11—C12	119.33 (13)	C19^i^—C19—H19B	109.0
C16—C11—C10	125.15 (14)	C18—C19—H19B	109.0
C12—C11—C10	115.52 (13)	H19A—C19—H19B	107.8
C13—C12—C11	120.82 (16)		
			
C4—N3—C2—N1	0.6 (3)	C6—N6—C10—O1B	−23.5 (8)
C6—N1—C2—N3	−0.5 (3)	C6—N6—C10—O1A	13.7 (14)
C2—N3—C4—N9	−179.85 (15)	C6—N6—C10—C11	173.08 (14)
C2—N3—C4—C5	−0.1 (2)	O1B—C10—C11—C16	−138.8 (8)
C8—N9—C4—N3	179.82 (16)	O1A—C10—C11—C16	−175.5 (14)
C8—N9—C4—C5	0.06 (18)	N6—C10—C11—C16	24.5 (3)
C8—N7—C5—C6	−179.15 (18)	O1B—C10—C11—C12	40.7 (9)
C8—N7—C5—C4	−0.01 (16)	O1A—C10—C11—C12	4.0 (14)
N3—C4—C5—N7	−179.79 (15)	N6—C10—C11—C12	−156.02 (16)
N9—C4—C5—N7	−0.03 (17)	C16—C11—C12—C13	−2.8 (3)
N3—C4—C5—C6	−0.4 (2)	C10—C11—C12—C13	177.68 (17)
N9—C4—C5—C6	179.31 (13)	C11—C12—C13—C14	1.8 (3)
C2—N1—C6—N6	178.40 (13)	C12—C13—C14—C15	0.0 (3)
C2—N1—C6—C5	−0.2 (2)	C13—C14—C15—C16	−0.7 (3)
C10—N6—C6—N1	−175.34 (15)	C12—C11—C16—C15	2.0 (3)
C10—N6—C6—C5	3.0 (3)	C10—C11—C16—C15	−178.52 (16)
N7—C5—C6—N1	179.62 (16)	C14—C15—C16—C11	−0.3 (3)
C4—C5—C6—N1	0.6 (2)	O3B—C17—C18—C19	26.0 (16)
N7—C5—C6—N6	1.3 (3)	O3A—C17—C18—C19	−19.2 (10)
C4—C5—C6—N6	−177.74 (14)	O2—C17—C18—C19	176.05 (16)
C4—N9—C8—N7	−0.08 (19)	C17—C18—C19—C19^i^	−176.11 (18)
C5—N7—C8—N9	0.06 (19)		

Symmetry code: (i) −*x*+1, −*y*+1, −*z*.

### Hydrogen-bond geometry (Å, º)

Cg is the centroid of the C11–C16 phenyl ring.

**Table:**

*D*—H···*A*	*D*—H	H···*A*	*D*···*A*	*D*—H···*A*
O2—H2*A*···N1^ii^	0.82	1.92	2.7327 (19)	175
N6—H6···O3*A*^iii^	0.86	2.09	2.904 (11)	157
N7—H7···O1*A*	0.86	2.04	2.616 (16)	124
N7—H7···N9^iv^	0.86	2.17	2.9271 (17)	146
C19—H19*B*···O3*A*^v^	0.97	2.54	3.481 (11)	164
C2—H2···*Cg*3^vi^	0.93	2.94	3.4611 (16)	117

Symmetry codes: (ii) *x*+1, *y*, *z*; (iii) *x*−1, *y*, *z*; (iv) −*x*+2, *y*+1/2, −*z*+1/2; (v) −*x*+2, −*y*+1, −*z*; (vi) *x*, *y*−1, *z*.
